# Preface

**DOI:** 10.1155/DTE.6.vii

**Published:** 2000

**Authors:** Yoshihiro Sakai


					Diagnostic and Therapeutic Endoscopy, Vol. 6, p. vii
Reprints available directly from the publisher
Photocopying permitted by license only
(C) 2000 OPA (Overseas Publishers Association) N.V.
Published by license under
the Harwood Academic Publishers imprint,
part of The Gordon and Breach Publishing Group.
Printed in Malaysia.
Preface
Cancer is the most life-threatening disease all over the world. To reduce cancer mortality, early detection
and treatment are essential. Endoscopy is the one of primary diagnostic methods for cancers of various
organs.
Initially endoscopic diagnosis was limited to organs which were located relatively near the body sur-
face: ear, nose, anus, etc. Due to the development of fiberoptic endoscopes, endoscopic examination
came to be performed in almost any space of the body. In addition to great progress in diagnosis, the
endoscopic treatment has begun in the earliest 1980's. Endoscopic resection has been the most common
method, especially in the field of gastroenterology. The improvements of accessory instruments as well as
in fiberscopes has made endoscopic treatment safer and less traumatic. Endoscopic resection is per-
formed safely using high frequency current. Furthermore, laser treatment has been increasingly common
employed in recent years. The indications of endoscopic treatment has increased particularly in recent
years and conventional open surgery is now sometimes avoidable. Endoscopists are continually develop-
ing new techniques depending on the site, size and shape of the tumors.
In this special issue, the latest techniques and results are presented by leading endoscopists. I hope for
that new developments through comparison of different techniques used in various fields.
Yoshihiro Sakai
Associate Editor
vii

				

